# Magneto‐Responsive Shutter for On‐Demand Droplet Manipulation

**DOI:** 10.1002/advs.202103182

**Published:** 2021-10-24

**Authors:** Jian Wang, Zhengxu Zhu, Pengfei Liu, Shengzhu Yi, Lelun Peng, Zhilun Yang, Xuelin Tian, Lelun Jiang

**Affiliations:** ^1^ Guangdong Provincial Key Laboratory of Sensor Technology and Biomedical Instrument School of Biomedical Engineering Sun Yat‐sen University Shenzhen 518107 China; ^2^ State Key Laboratory of Optoelectronic Materials and Technologies School of Materials Science and Engineering Sun Yat‐sen University Guangzhou 510006 China; ^3^ Key Laboratory for Polymeric Composite and Functional Materials of Ministry of Education Guangzhou Key Laboratory of Flexible Electronic Materials and Wearable Devices Sun Yat‐sen University Guangzhou 510006 China

**Keywords:** droplet transport, intelligent surfaces, laser kirigami, magnetic actuation, wettability

## Abstract

Magnetically responsive structured surfaces enabling multifunctional droplet manipulation are of significant interest in both scientific and engineering research. To realize magnetic actuation, current strategies generally employ well‐designed microarrays of high‐aspect‐ratio structure components (e.g., microcilia, micropillars, and microplates) with incorporated magnetism to allow reversible bending deformation driven by magnets. However, such magneto‐responsive microarray surfaces suffer from highly restricted deformation range and poor control precision under magnetic field, restraining their droplet manipulation capability. Herein, a novel magneto‐responsive shutter (MRS) design composed of arrayed microblades connected to a frame is developed for on‐demand droplet manipulation. The microblades can perform two dynamical transformation operations, including reversible swing and rotation, and significantly, the transformation can be precisely controlled over a large rotation range with the highest rotation angle up to 3960°. Functionalized MRSs based on the above design, including Janus‐MRS, superhydrophobic MRS (SHP‐MRS) and lubricant infused slippery MRS (LIS‐MRS), can realize a wide range of droplet manipulations, ranging from switchable wettability, directional droplet bounce, droplet distribution, and droplet merging, to continuous droplet transport along either straight or curved paths. MRS provides a new paradigm of using swing/rotation topographic transformation to replace conventional bending deformation for highly efficient and on‐demand multimode droplet manipulation under magnetic actuation.

## Graphical abstract

A novel magneto‐responsive shutter (MRS) design is put forward for intelligent droplet manipulation. Different from conventional strategies relying on bending deformation of high‐aspect‐ratio microstructures, MRS employs swing/rotation transformation and realizes large deformation with high control precision, thus enabling on‐demand droplet manipulation, ranging from switchable wettability, directional droplet bounce, to continuous droplet transport along either straight or curved paths.

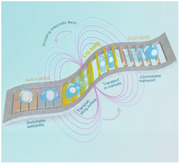

## 1 Introduction

Dynamical droplet manipulation using responsive structured surfaces has attracted a lot of attentions due to their great potential in water collection,^[^ ^1^, ^2^, ^3^ ^]^ digital microfluidics,^[^ ^4^, ^5^ ^]^ chemical reaction,^[^ ^6^, ^7^ ^]^ and biomedical analysis.^[^ ^8^, ^9^ ^]^ Responsive structured surfaces can reversibly change the surface topography in response to external stimuli (e.g., mechanical forces,^[^ ^10^, ^11^, ^12^ ^]^ electric,^[^ ^5^, ^13^ ^]^ thermal,^[^ ^14^ ^]^ light,^[^ ^8^, ^9^, ^15^ ^]^ and magnetic field).^[^ ^16^ ^]^ Among these manipulation strategies, magnetic actuation has the incomparable advantages of biocompatibility, remote control, instantaneous response, and free of specific environmental requirement.^[^ ^17^, ^18^, ^19^ ^]^ As a result, magneto‐responsive structured surfaces (MRSS) are particularly suitable for active droplet manipulation, such as switchable wettability,^[^ ^20^, ^21^ ^]^ directional droplet transportation,^[^ ^6^, ^7^, ^22^ ^]^ reversible droplet adhesion,^[^ ^2^, ^23^ ^]^ and fog collection.^[^ ^1^, ^3^ ^]^

MRSS with various structured topographies including conical array, microcilia array,^[^ ^1^, ^2^, ^7^, ^23^, ^24^ ^]^ micropillars,^[^ ^25^, ^26^, ^27^, ^28^, ^29^, ^30^, ^31^ ^]^ and microplates have been designed for dynamical droplet manipulation under magnetic actuation.^[^ ^6^, ^21^, ^32^, ^33^ ^]^ For example, magneto‐responsive conical array was employed for static fog collection under windless regions.^[^ ^1^ ^]^ Magneto‐responsive arrays of microcilia and micropillars can realize switchable wettability,^[^ ^2^, ^20^, ^23^ ^]^ reversible adhesion,^[^ ^31^ ^]^ directional droplet transportation,^[^ ^7^, ^22^, ^26^, ^34^, ^35^ ^]^ and droplet bouncing.^[^ ^24^ ^]^ MRSS with magneto‐responsive Janus microplates enabled reversible switch between superhydrophobic and hydrophilic states,^[^ ^21^ ^]^ and achieved 3D droplet transport.^[^ ^6^ ^]^ In the above MRSS, their surface microstructures undergo large bending under an oblique magnetic field, resulting in formation of switchable microstructured surfaces with anisotropic wettability for dynamic droplet manipulation.^[^ ^2^, ^20^, ^21^, ^23^ ^]^ To lower the structure stiffness, MRSS always utilize magneto‐responsive microstructures with large aspect ratios to allow bending deformation.^[^ ^1^, ^28^, ^29^, ^36^ ^]^ However, such magneto‐responsive bendable microstructures suffer from highly restricted deformation range as well as single transformation mode, which severely limit the multifunctional droplet manipulation capability. Moreover, it is also difficult to accurately control the bending degree of the microstructures in a predesigned way, resulting in failure of high‐precision liquid manipulation. Therefore, it is in high demand to develop novel design strategies of MRSS capable of multimodal transformation for on‐demand droplet manipulation with high control precision.

Herein, we put forward a novel magneto‐responsive shutter (MRS) design for on‐demand and multifunctional droplet manipulations. MRS is extremely simple in structure and is only composed of microblade array connected to a frame with two cords. Functionalized MRSs based on the above design, including Janus‐MRS, superhydrophobic MRS (SHP‐MRS), and lubricant infused slippery MRS (LIS‐MRS) were fabricated from a magneto‐active soft membrane using facile techniques of laser kirigami and surface modification. Two tunable transformation behaviors, that is, reversible swing and rotation, with precisely controllable swing/rotation angles, were realized for MRSs under simple magnetic actuation. The transformation mechanism of MRS under magnetic actuation was attributed to the quasi‐static balance between magnetic torque, *T*_m_, and elastic shear torque, *T*_s_. Based on the precise control of transformation behaviors of MRS, a variety of droplet manipulations were successfully achieved, including rapid switchable wettability, directional droplet bounce, droplet penetration and distribution, droplet merging, and continuous droplet transport along either straight or curved paths. MRS shows significant advantages both in fabrication strategy and on‐demand droplet manipulation capability over existing works (Details in **Table** **1**). This work opens a new avenue for multifunctional droplet manipulations in a highly controllable way and extends the functional applications of MRSS.

**Table 1:** Comparison between this work and existing MRSS in literature

	Fabricate strategy [difficulty]	Deformation	Droplet manipulation	Reference
Microcilia	Magnetic self‐assembly (Intermediate)	Bending	Undirectional wettability	^[^ ^23^ ^]^
			Reversible adhesion	^[^ ^2^ ^]^
			Fog collection	^[^ ^3^ ^]^
			Directional bouncing	^[^ ^24^ ^]^
	Replica‐molding (Sophisticated)		Fog collection	^[^ ^1^ ^]^
			Droplet transportation	^[^ ^7^ ^]^
Micropillar	Replica‐molding (Sophisticated)		Directional transportation	^[^ ^28^ ^]^
			Droplet capture	^[^ ^36^ ^]^
			Droplet transportation	^[^ ^26^ ^]^
Microplate	Replica‐molding (Sophisticated)		Switchable wettability	^[^ ^21^ ^]^
			Droplet transportation	^[^ ^6^ ^]^
			Switchable adhesion	^[^ ^33^ ^]^
MRS	Laser kirigami (Facile)	1. Swing 2. Rotation	Switchable wettability Directional bouncing Droplet distribution Droplet merging Droplet transportation	This work

## 2 Results and Discussion

**Figure 1:**
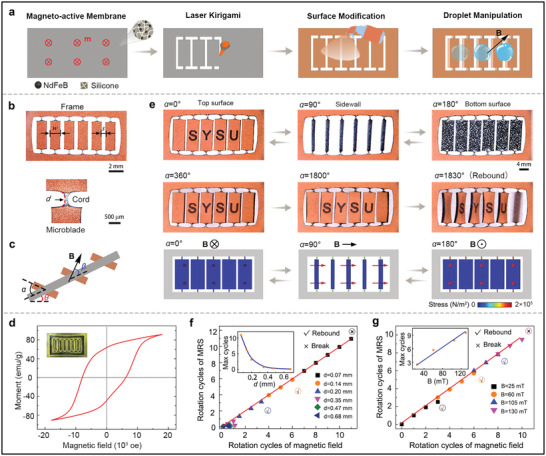
Fabrication and magnetic actuation behaviors of MRS. a) Illustration of the fabrication process of MRS. b) Images of the prestretched MRS. *w* and *s* are the width and the interval of microblades, respectively. *d* is the diameter of the cord. c) Schematic illustration of MRS rotation under magnetic actuation. *α* and *β* are the rotation angles of MRS and actuating magnetic field, respectively. *θ* is the tilt angle of MRS. d) Magnetic hysteresis loop of MRS. Insert is the magnetization profile of MRS. e) Images and FEA results of the swing and rotation process of MRS under magnetic actuation. f) The maximum rotation cycles of MRS with *d* of 0.07, 0.14, 0.20, 0.35, 0.47, and 0.68 mm under 60 mT magnetic actuation. g) The maximum rotation cycles of MRS with *d* of 0.14 mm under actuating field intensity of 25, 60, 105, and 130 mT.

MRS was fabricated by a facile process of laser kirigami and surface modification (**Figure** **1**a). A large magneto‐active soft membrane with a homogeneous magnetization profile **m** was prepared using spin coating and magnetizing according to a previously reported method.^[^ ^37^ ^]^ One side of the magneto‐active soft membrane was uniformly coated with copper nanoparticles to help distinguish the top and the bottom surfaces. Based on predesigned laser scanning path, numerous microblades were cut out from the magneto‐active soft membrane to get a MRS using laser kirigami technology. Subsequently, the top and bottom surfaces of MRS were modified into superhydrophobic or slippery state. The copper nanoparticle coating has negligible effect on the superhydrophobicity and slippery performance of MRS. This fabrication process is cost‐effective, highly efficient, and suitable for large‐scale manufacturing. The fabricated MRS in prestretched state is shown in Figure 1b,c (Figure S1, Supporting Information). The microblades are orderly arranged and connected to the frame with two slender cords. MRS is mainly composed of silicone embedded with hard‐magnetic neodymium‐iron‐boron (NdFeB) microparticles. Silicone with excellent ductility and NdFeB microparticles with the strongest hard‐magnetic property is combined to yield good elasticity (Figure S2, Supporting Information) and strong hysteresis (Figure 1d). The Young's modulus and remnant magnetization of MRS were measured to be 194 KPa and 65 emu g^−1^, respectively. The magnetization profile of MRS was visualized using a magnetic indicator (Figure 1d), showing the uniform magnetic field distribution in the microblades.

Two transformation behaviors of MRS, including reversible swing or rotation, could be realized under magnetic actuation. The transformation behaviors of MRS microblades driven by magnetic field B were experimentally investigated and numerically calculated, as shown in Figure 1e (Figure S3 and Video S1, Supporting Information). In the swing mode, the microblades swing back and forth under a pendular actuating field. As the actuating field B gradually turned from 90° to −90°, the microblades synchronously rotated from 0° to 180°. The rotation angles of MRS can be accurately controlled by the external magnetic field B, as shown in Figure S4, Supporting Information. The accuracy of the rotation/swing angle is determined by the rotation behavior of the magnetic field. In the rotation mode, the rotating magnetic field actuates the microblades to synchronously rotate for multiple circles. The simulated rotation process of MRS under magnetic actuation is well consistent with the experimental results (Video S2 and Figure S5, Supporting Information). The simulation also show that the principal stress increased constantly and centralized in the slender cords during the rotation process (Figure 1e).

A microblade can be regarded as a magnet, and two torques, including the magnetic torque, *T*_m_, and elastic shear torque, *T*_s_, act on the microblades during swing/rotation. Once actuating field **B** is applied, a magnetic torque *T*_m_ is produced due to the interaction between **B** and magnetic profiles **m**:^[^ ^21^, ^38^ ^]^

(1) $$T_{m}=\underset{0}{\int^{V_{m}}}mB\mathrm{dv}$$

where *V*_m_ is the total volume of a microblade. According to the principle of minimum potential energy, the microblades tend to align their magnetization profile **m** along the direction of the actuation field **B**.^[^ ^37^ ^]^ Therefore, the magnetic torque *T*_m_ leads to a rotation or swing of the microblade. According to Hooke's law, the elastic shear torque, *T*_s_, is attributed to the rotation‐induced deformation of microblades, which can be given by Equation (2):

(2) $$T_{s}=\frac{G\pi d^{4}\alpha }{32}\;$$

where *G* is the shear modulus of magneto‐active soft membrane, *d* is the diameter of the junction cord, and *α* is the rotation angle of the microblades. According to Equation (2), the rotation‐induced shear torque, *T*_s_, increases with rotation angle *α*, resisting MRS rotation process. The resistance of *T*_s_ produces a hysteresis angle, *γ*, between the actuating field **B** and magnetization profiles **m** of the microblades. According to Equation (1), the magnetic torque *T*_m_ increases with hysteresis angle *γ*, which accordingly drives the rotation of MRS. Both the magnetic torque, *T*_m_, and the rotation‐induced shear torque, *T*_s_, increase during the MRS rotation process. Therefore, the interaction between magnetic torque, *T*_m_, and the rotation‐induced shear torque, *T*_s_, can reach a dynamic balance, that is, *T*_m_ equals to *T*_s_. The rotation angle or swing angle, *α*, can be precisely controlled by the magnetic field **B**.

However, there are two cases that the rotation process may get failed. First, the rotation‐induced shear torque, *T*_s_, increases with rotation angle *α*, which is beyond the shear fracture limit of MRS, resulting in the fracture failure at the cords. Second, the hysteresis angle *γ* between the actuating field **B** and magnetization profiles **m** increases with the rotation, which gradually gets close to 90°. The magnetic torque reaches the maximum value as *γ* ≈ 90°, but fails to drive the further rotation of MRS due to the large shear torque, resulting in the resilience of the microblades. Therefore, there exists a maximum rotation cycle for a given MRS under a certain magnetic actuation (Figure 1e and Figure S3c, Supporting Information).

The diameter of the junction cords and the actuating field intensity play an important role in the rotation behavior of MRS. The maximum rotation cycles of MRS decreases with the cord diameter *d* (Figure 1f). When *d* = 0.07 mm, MRS can rotate for ≈11 cycles (almost 3960°), and then the junction cords is finally broken due to the extremely high rotation‐induced shear stress focused on the cords. When *d* = 0.14 mm, the maximum rotation cycles is six (almost 2160°) without break of junction cords and then MRS rebounds. The effect of magnetic field intensity on the rotation behavior of MRS is shown in Figure 1g. The maximum rotation cycles of MRS increase (from ≈2 to 10) with the actuating field intensity (from 25 to 130 mT). The MRS with cords diameter of 0.14 mm and actuating field intensity of 105 mT was chosen for the following investigations.

**Figure 2:**
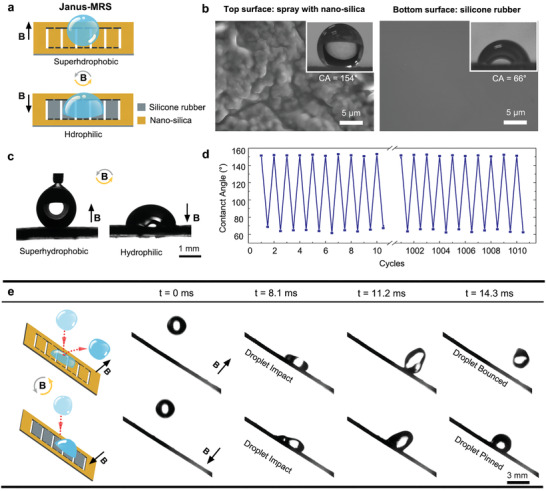
Janus‐MRS with switchable wettability. a) Schematic of Janus‐MRS with switchable wettability under magnetic actuation. b) SEM images and corresponding water contact angles of the top surface and the bottom surface, respectively. c) Optical images demonstrate magneto‐responsive switchable wetting states between superhydrophobicity and hydrophilicity of Janus‐MRS (3 µL). d) The wetting stability of Janus‐MRS for 1000 cyclic switches under magnetic actuation. e) The droplet (9 µL) impact behaviors on Janus‐MRS with switchable wettability. The water droplet is bounced away from the superhydrophobic side while pinned on the hydrophilic side upon magnetic actuation.

Simple surface functionalization of MRS can facilitate its applications in various modes of droplet manipulations. We first prepared Janus‐MRS with switchable wettability, as illustrated in **Figure** **2**a. The bottom surface without modification is smooth with a water contact angle of about 66°, and the top surface coated with a homemade Nano‐SiO_2_ spray featuring hierarchical micro‐/nano‐structures is superhydrophobic with a water contact angle of ∼154° (Figure 2b and Figure S6, Supporting Information). Janus‐MRS can arbitrarily switch between its superhydrophobic surface and hydrophilic one via rotating of magnetic field by 180° (Figure 2c and Video S3, Supporting Information). Such magneto‐responsive wettability switch between superhydrophobicity and hydrophilicity is rapid, and notably, can be repeated for more than 1000 cycles without observable performance degradation (Figure 2d).

Janus‐MRS also showed switchable droplet impact behaviors (Figure 2e and Video S4, Supporting Information). On a 45° inclined Janus‐MRS, a water droplet impacting the surface would bounce up with a contact time of about 14.3 ms when the superhydrophobic surface faced upward, indicating a low‐adhesion water repellent state. However, when the hydrophilic surface was turned upward under magnetic actuation, a water droplet impacting the surface would immediately get pinned on the surface. That is, Janus‐MRS can be utilized for on‐demand selective bounce or capture of water droplets under magnetic actuation.

**Figure 3:**
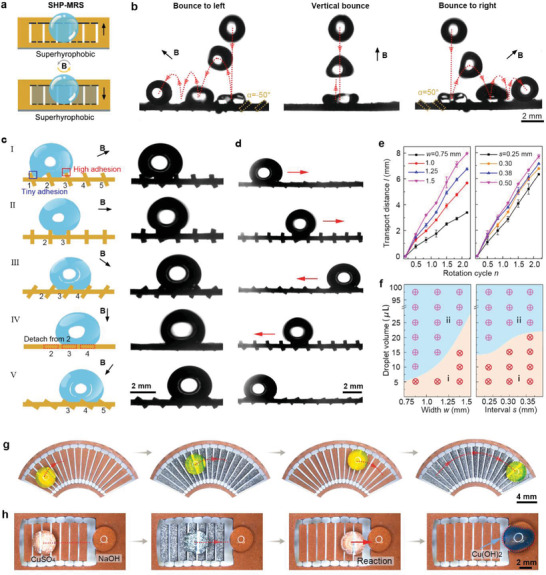
Directional bounce and controllable transport of droplets on SHP‐MRS. a) Schematic of SHP‐MRS under magnetic actuation, whose surfaces are superhydrophobic. b) The bounce behaviors of droplets (6 µL) on SHP‐MRS at swing angles *α* of −50°, 0°, and 50°, respectively. c) The directional transport of water droplet (18 µL) on SHP‐MRS (*w* = 1.25 mm, and *s* = 0.30 mm) under a rotating magnetic field. d) The droplet (18 µL) moves back and forth on SHP‐MRS driven by the clockwise and anticlockwise rotating magnetic field, respectively. e) The effects of rotation cycles *n* on the transport distance, *l*, under *w* of 0.75, 1.0, 1.25, and 1.5 mm and *s* of 0.25, 0.30, 0.38, and 0.50 mm, respectively. f) The phase diagram revealing the transportation capability of SHP‐MRS under different widths and intervals of the microblades. The region (i) represents the failure of droplet transport in which droplets gets stuck between the intervals of the microblades, while the region (ii) represents the success of droplet transport. g) The fan‐shaped SHP‐MRS (*w* = 0.65–1.4 mm,and *s* = 0.30–0.50 mm) delivers the droplet (30 µL) in an arc pathway. h) Demonstration of SHP‐MRS (*w* = 1.25 mm, and *s* = 0.30 mm)‐ based microreactor. The CuSO_4_ droplet (30 µL) is delivered to react with NaOH droplet, and blue Cu(OH)_2_ precipitates are synthesized.

We next prepared SHP‐MRS by modifying both sides of the microblades with nano‐SiO_2_ spray coating, which enabled controllable directional bounce and transport of water droplets under magnetic actuation (**Figure** **3**a). The surfaces of SHP‐MRS exhibited superhydrophobicity with teeny adhesion (Figures S7 and S8, Supporting Information). The droplet bounce behaviors on the SHP‐MRS at swing angles *α* of −50°, 0°, and 50° are shown in Figure 3b (Figure S9 and Video S5, Supporting Information). When a droplet fell down and had hit on the microblades with *α* = 0°, vertical bounce was observed. When a droplet hit the tilted microblades with *α* = −50° (50°), the droplet got rebounded toward the left (right) attributed to the unbalanced rebound force induced by the tilted microblades (droplet‐seesaw effect).^[^ ^24^, ^39^ ^]^ Therefore, the bounce direction of droplet can be easily tuned via the swing angle of microblades under magnetic actuation.

The controllable directional transport of water droplet can be realized using continuous rotation of the microblades under magnetic actuation, as shown in Figure 3c (Video S6, Supporting Information). The droplet transport process can be divided into five typical stages: I) When the microblades begin to rotate at a rotation angle of *α *= 45°, the droplet sits on the microblades 1, 2, and 3. II) When the microblades clockwise rotate at *α* = 90°, the droplet is pulled by the sidewall of microblade 3 and detached from microblade 1 owing to the high adhesion of sidewall and the low adhesion of top surface (Figures S7 and S8, Supporting Information). III) When the microblades continuously clockwise rotate at *α* = 135°, the droplet is driven by microblades 2 and 3 to the right, and contacts with microblade 4. IV) When the microblades clockwise rotate at *α *= 180°, the droplet detaches from the sidewall of microblade 2 and sits on the bottom surface of microblade 3 and 4 due to the shear effect of the microblades on droplet. V) When the microblades further clockwise rotate, the above process would be repeated. The droplet transport distance per rotation cycle is 2(*w* + *s*). By repeating above stages, the droplet can be continuously transported forward. The mass loss of liquid can be ignored during the manipulation process due to the extremely low adhesion of the droplet on the SHP‐MRS. Moreover, the droplet can move back and forth by switching the rotation direction of the magnetic field, as shown in Figure 3d (Video S6, Supporting Information). The droplet moves forward under a clockwise rotating magnetic field, and moves backward under an anticlockwise rotating magnetic field. The effects of rotation cycles *n*, the width *w*, and intervals *s* on the droplet transport distance *l* of SHP‐MRS are shown in Figure 3e (Figures S10 and S11, Supporting Information). The droplet transport distance *l* almost linearly increases with the rotation cycles *n*, that is *l* = 2*n*(*w* + *s*). The droplet volume affects little on the transport distance (Figure S12, Supporting Information). The droplet transport speed is mainly determined by the swing/rotation speed of the MRS, which is directly actuated by the external magnetic field. The average droplet transport speed along the straight (Figure 3d) and the curved (Figure 3g) path is 0.85 and 0.79 mm s^−1^, respectively. The transport speed almost linearly increases with the rotation speed *m*_r_ (rotation cycles per second), that is *v* = 2*m*_r_(*w* + *s*).

It should be noted that only droplets above a certain size can be transported along SHP‐MRS. Figure 3f shows the critical volume of transportable droplets on SHP‐MRS with different widths and intervals. Droplets of small size would get stuck in the intervals of the microblades, leading to the failure of transport (Figure 3f, Region i). The critical volume of transportable droplets increase with the width, *w*, and interval, *s*, of the microblades (Figure 3f, Region ii). Furthermore, SHP‐MRS is able to transport ultralarge droplet (≥100 µL, Figure S13, Supporting Information).

Besides transport along a straight path, SHP‐MRS can also realize droplet transport along a curved path. As shown in Figure 3g (Video S7, Supporting Information), a fan‐shaped SHP‐MRS delivers a droplet in a particular arc path, demonstrating the versatility of MRS‐SHP in intelligent droplet transport. Moreover, SHP‐MRS can be used as a miniature reactor for controllable chemical reaction. As demonstrated in Figure 3h (Video S7, Supporting Information), a CuSO_4_ droplet is transported toward a NaOH droplet under magnetic actuation. Once they are merged together, Cu(OH)_2_ precipitates are formed.

**Figure 4:**
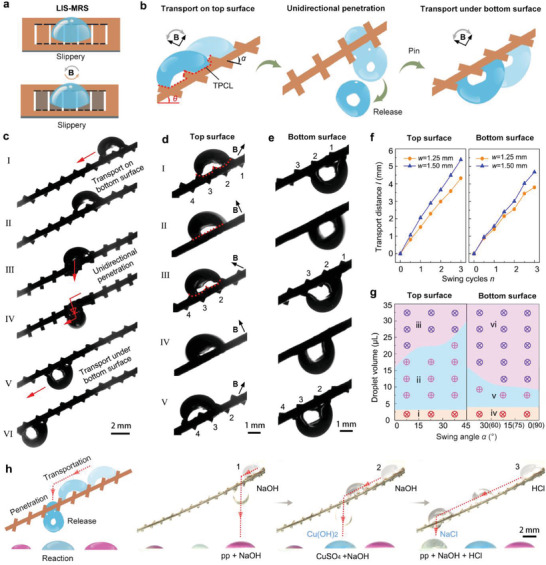
Multifunctional droplet manipulation using a slant LIS‐MRS. a) Schematic of LIS‐MRS under magnetic actuation, whose surfaces are slippery. b) Schematic illustration of droplet transport on the top surface, penetration through interval of microblades, and transport under the bottom surface of the slant LIS‐MRS. c) The continuous droplet (7 µL) delivery process. It is composed of the droplet transport on the top surface, the droplet penetration, and the droplet transport under the bottom surface of a slant LIS‐MRS (*w* = 1.25 mm and *s* = 0.30 mm). d,e) Five typical stages of droplet transport on d) the top surface and e) the bottom surface of slant LIS‐MRS, respectively. f) The relationship between droplet transport distance, *l*, and swing cycles, *n*, of actuating field. The widths of microblades, *w*, are 1.25 and 1.5 mm, respectively. g) The phase diagram revealing droplet transport behaviors of slant LIS‐MRS under different swing angles *α*. The regions (i) and (iv) represent that small droplets get pinned on the surfaces. The regions (ii) and (v) represent that the droplet can be successfully transported along LIS‐MRS. The region (iii) represent that the large droplet cannot be transported along LIS‐MRS. The region (vi) represents the droplet may fall off from LIS‐MRS. h) Droplet (12 µL) distribution using LIS‐MRS (*w* = 1.50 mm and *s* = 0.30 mm) for programmable chemical reaction. Droplets were one by one transported to the targeted positions on LIS‐MRS and distributed to react with the underlying droplets.

We further prepared LIS‐MRS by coating the pristine MRS surfaces with a thin layer of silicone oil (**Figure** **4**a). The *Nepenthes*‐inspired LIS surface is known to have excellent water repellency.^[^ ^40^ ^]^ Both surfaces of LIS‐MRS exhibit excellent slippery characteristics, with water contact angle and sliding angle of ≈86° and 10° (≈5 µL), respectively (Figures S7 and S14, Supporting Information). LIS‐MRS can realize three typical dynamic droplet manipulations by swinging of the actuating field: transport on the top surface, controllable penetration through the interval, and transport along the bottom surface, as demonstrated in Figure 4b. A continuous droplet transport process on a slant LIS‐MRS (slant angle ≈ 30°), composed of transport on top surface, directional penetration, and transport along bottom surface, is presented in Figure 4c (Video S8, Supporting Information). The water droplet is delivered on the top surface interval‐by‐interval via periodical swing of the microblades (I and II). When transferred to a target position, it is allowed to penetrate through the interval between two microblades by increasing the swing angle *α* (III and IV). Subsequently, the penetrated droplet is hanged by the microblades and transported along the bottom surface via periodical swing of magnetic field (V and VI). It should be noted that a minor amount of mass loss of droplet can be observed during the manipulation process because the LIS‐MRS is slightly adhesive to the droplet.

The detailed droplet transport process on the top surface is illustrated in Figure 4d (Figure S15, Supporting Information), which can be divided into five stages. I) When the swing angle *α* of the microblades is 0° < *α* < 45°, the droplet is pinned on the microblades 1, 2, and 3. II) When the microblades are clockwise swung to *α* = 0°, resulting in an unstable state of droplet due to the rapid decrease of the triple‐phase contact line (TPCL). The droplet detached from microblade 1 and then moves to the microblades 2 and 3. III) When the microblades are continuously clockwise rotated with −45° < *α* < 0°, the droplet slips down and is pined on the microblades 2, 3, and 4, tending to a more stable state of droplet due to the increase of TPCL and release of surface energy.^[^ ^41^ ^]^ IV) When the microblades are anticlockwise rotated to *α* = 0°, the droplet moves to the microblades 2, 3, and 4. V) When the microblades are anticlockwise rotated to *α* (0° < *α* < 45°), the droplet rapidly slips down and is pinned on the microblades 2, 3, and 4, leading to a more stable state of droplet due to the increase of TPCL. The droplet transport along the bottom surface of LIS‐MRS is similar to that on the top surface, as shown in Figure 4e (Figure S15, Supporting Information). The droplet transport distance per swing cycle is *w* + *s*. The droplet transport distance *l* along the top or the bottom surface almost linearly increases with the swing cycles *n* of actuating field, that is *l* = *n*(*w* + *s*), as shown in Figure 4f (Figure S16, Supporting Information), and the droplet volume affects little on the transport distance (Figures S17–S19, Supporting Information). The average droplet transport speed is 0.92 mm s^−1^ (Figure 4c–e) and the droplet transport speed *v* almost linearly increases with the swing speed *m*_s_ (swing cycles per second), that is *v* = *m*_s_(*w* + *s*). Therefore, the slant LIS‐MRS can precisely transport droplets interval‐by‐interval by periodic swing of actuating field compared to transport on previously reported lubricant infused surfaces.^[^ ^8^, ^12^, ^42^ ^]^

The effects of swing angle, *α*, and droplet volume on the transport behaviors along the top and bottom surfaces of slant LIS‐MRS was investigated (Figure 4g and Figure S20, Supporting Information). Too small droplets would stand still on the top surface (Region i) or the bottom surface (Region iv) due to insufficient driven force of gravity. Droplets of too large size would rapidly slip down from the top surface (Region iii) or fall off from the bottom surface (Region vi) due to the dominant role of large gravity relative to droplet adhesion. Only droplets in appropriate sizes can be transported along the top surface (3–30 µL, Region ii) or the bottom surface (4–16 µL, Region v). Increasing the swing angle *α* (<45°) can enhance the transport ability both on the top and bottom surfaces. However, the droplet may penetrate through the interval of the microblades once the swing angle *α* exceeds 45° and get stuck by the bottom surface (Regions iv and v) or fall off from LIS‐MRS (Region vi). The volume ranges suitable for droplet transport are maximized at intermediate slant angles, *θ*, of LIS‐MRS, which are around 20° and 30° for the top and the bottom surfaces, respectively (Figure S21, Supporting Information). This is due to that small slant angle could not provide sufficient driving force for droplet motion and large slant angle would result in droplet slipping down or falling off the surfaces.

As mentioned above, the droplet can penetrate through the intervals of the microblades once the swing angle *α* goes beyond 45°, and large droplets (Region vi) would fall off from LIS‐MRS as the gravity component can overcome droplet adhesion, which can be used for controlled droplet release. The droplet transport on the top surface and droplet release can be combined together for LIS‐MRS‐based droplet distribution under magnetic actuation, and employed to construct magnetically responsive chemical reaction platform. As shown in Figure 4h (Video S9, Supporting Information), one by one, three droplets (NaOH, NaOH, and HCl) are accurately transported to the target positions, penetrate through the intervals of microblades, and are released to react with the underlying droplets (phenolphthalein [pp], CuSO_4_, and pp + NaOH). The precise droplet distribution ability of LIS‐MRS using swing operation exhibits promising application potential in magneto‐responsive programmable chemical reactions.

**Figure 5:**
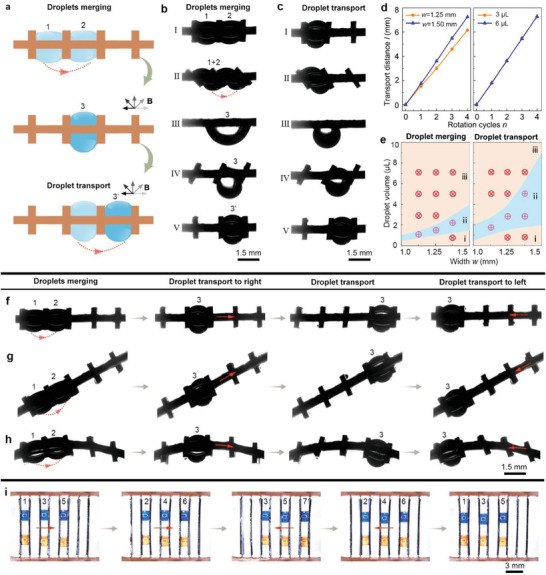
Droplets merging and droplet transport in the intervals of LIS‐MRS (*w* = 1.5 mm, *s* = 0.30 mm) using rotation actuation. a) Schematic diagram of droplets merging and droplet transport in the intervals of LIS‐MRS driven by a rotating magnetic field B. b) The detailed droplets (3 µL) merging process of LIS‐MRS. c) The detailed droplet transport process of LIS‐MRS. d) The effects of rotation cycles *n* on the droplet transport distance, *l*, under *w* of 1.25 and 1.5 mm and droplet volume of 3 and 6 µL, respectively. e) The phase diagram revealing droplets merging and droplet transport behaviors of LIS‐MRS under different microblade widths, *w*. The region (i) represents that the droplet is adhered to one microblade and cannot contact the adjacent one, the region (iii) represents that the droplet is too large to be delivered by the microblade. The region (ii) represents that the droplet can be successfully merged and delivered. The droplets merging and droplet transport back and forth along f) a horizontal LIS‐MRS, g) a slant LIS‐MRS, and h) a curved LIS‐MRS, respectively. i) The transport of droplet (4 µL) array along a LIS‐MRS.

Besides swing actuation, LIS‐MRS can employ rotation actuation to achieve droplet merging and droplet transport manipulations (**Figure** **5**a). Two adjacent droplets are merged as the microblades are continuously rotated, as shown in Figure 5b (Figure S22 and Video S10, Supporting Information). The droplets merging process can be divided into five stages. I) Two droplets (droplet 1 and droplet 2) are sandwiched in the adjacent intervals of microblades. II) When the microblades are anticlockwise rotated, the droplets are gradually extruded by the microblades. III) Two extruded droplets contact with each other and merge into a bigger one (droplet 3), which is hanged by the bottom surface when the microblades are rotated at *α* = 0°. IV) When the microblades are continuously rotated, droplet 3 is transferred to the second interval, and V) when the microblades are rotated at *α* = 90°, droplet 3′ is sandwiched in the next interval.

A droplet can also be transported from one interval to the next interval by continuous rotation of the microblades, as shown in Figure 5c (Figure S22 and Video S10, Supporting Information). This process can also be divided into five stages. I) When the rotation angle is *α* = −90°, the droplet is sandwiched in the first interval. II) When the microblades are anticlockwise rotated, the droplet is gradually extruded, and III) when the microblades are rotated at *α* = 0°, the extruded droplet is hanged by the bottom surface of the second microblade. IV) When the microblades are continuously rotated, the droplet is transferred to contact with the third microblade. V) When the microblades were rotated at *α* = 90°, the droplet is sandwiched between the second and third microblades due to the release of surface energy. The transport distance per rotation cycle is *w* + *s*. By repeating the above stages, the droplet can be continuously transported interval by interval. The droplet transport distance *l* linearly increases with the rotation cycles *n*, as shown in Figure 5d (Figures S23 and S24, Supporting Information), and the droplet volume shows negligible effect on the transport distance (Figure S25, Supporting Information). The average droplet transport speed is 0.69 mm s^−1^ (Figure 5b,c) and the droplet transport speed *v* almost linearly increases with the rotation speed *m*_r_, that is *v* = *m*_r_(*w* + *s*).

The effects of droplet volume and width, *w*, of microblades on the droplet merging and transport ability were investigated (Figure 5e). Droplets of small size fail to be merged or transported (Region i) as they are adhered to one microblade without contacting with the adjacent one. On the other hand, droplets of too large size cannot be carried to the next microblade at stage IV due to their large gravity component, and are also unable to be merged or transported (Region iii).

Figure 5f (Video S11, Supporting Information) demonstrates droplet merging, and subsequent back‐and‐forth transport in the intervals of horizontal LIS‐MRS by switching the rotation direction of magnetic field. LIS‐MRS can enable droplet merging and transport along an upward slant path with the slant angle, *θ*, of ≈30°, demonstrating its antigravity transport ability under rotating magnetic actuation (Figure 5g and Video S11, Supporting Information). The volume range appropriate for antigravity transport along a slant LIS‐MRS decreases with the slant angle, *θ*, as large *θ* leads to increased tangential gravity component that resists uphill droplet transport (Figure S26, Supporting Information). Even when the flexible LIS‐MRS was curved, the droplets can still be successfully merged and transported, as shown in Figure 5h (Video S11, Supporting Information). The droplet also can be transported in a particular arc path using a fan‐shaped LIS‐MRS (Figure S27 and Video S11, Supporting Information). Furthermore, LIS‐MRS can be employed for synchronous transport of droplet array under magnetic actuation, as shown in Figure 5i (Video S12, Supporting Information). The droplet array (2 × 3) is transferred back and forth interval by interval using the LIS‐MRS. All the above results manifest the excellent versatility of LIS‐MRS in intelligent droplet transport.

## 3 Conclusions

In summary, we put forward a novel MRS design for on‐demand and multifunctional droplet manipulation. Janus‐MRS, SHP‐MRS, and LIS‐MRS were fabricated via the integrated process of laser kirigami and surface modification. Unlike conventional magnetic actuation strategies which generally utilize bending deformation of high‐aspect‐ratio microstructure components to manipulate droplet behaviors, two unique transformation behaviors of MRS, including reversible swing and rotation, are employed for on‐demand droplet manipulations. Multiple intelligent droplet manipulations, including switchable wettability, directional droplet bounce, controllable droplet penetration, droplet distribution, droplets merging, and continuous droplet transport along either straight or curved paths, are successfully achieved using MRS under magnetic actuation. The multifunctional MRS offers a new design paradigm of magnetically responsive structured surfaces enabling intelligent and versatile droplet manipulation, which could bring promising applications in microfluidics,^[^ ^4^, ^5^ ^]^ programmable chemical reaction,^[^ ^6^, ^7^ ^]^ biological analysis,^[^ ^8^, ^9^ ^]^ medical diagnosis, and biomedical devices.^[^ ^43^, ^44^ ^]^ In addition, we anticipate that MRS will benefit areas such as tunable optical devices,^[^ ^21^, ^38^ ^]^ and solar cell.^[^ ^45^ ^]^

## Conflict of Interest

The authors declare no conflict of interest.

## Supporting information

Supporting Information

Supplemental Video 1

Supplemental Video 2

Supplemental Video 3

Supplemental Video 4

Supplemental Video 5

Supplemental Video 6

Supplemental Video 7

Supplemental Video 8

Supplemental Video 9

Supplemental Video 10

Supplemental Video 11

Supplemental Video 12

## Data Availability

Research data are not shared.
