# Supplementary material for: Magneto‐Responsive Shutter for On‐Demand Droplet Manipulation
Source: Adv Sci (Weinh). 2021 Oct 24;8(23):2103182. doi: 10.1002/advs.202103182 (PMC8655205; doi:10.1002/advs.202103182)
Supplement: Supplementary file 1 — Supporting Information [file ADVS-8-2103182-s010.pdf]

© 2021 Wiley-VCH GmbH

ADVANCED SCIENCE
Open Access

# Supporting Information

for *Adv. Sci.*, DOI: 10.1002/advs.202003182

# Magneto-Responsive Shutter for On-Demand Droplet Manipulation

*Jian Wang, Zhengxu Zhu, Pengfei Liu, Shengzhu Yi, Lelun Peng, Zhilun Yang, Xuelin Tian*, Lelun Jiang**

# Supporting Information

## Magneto-Responsive Shutter for On-Demand Droplet Manipulation

*Jian Wang, Zhengxu Zhu, Pengfei Liu, Shengzhu Yi, Lelun Peng, Zhilun Yang, Xuelin Tian*, Lelun Jiang**

## 1. Experimental Section

### 1.1 Preparation of MRS

Silicone pre-polymer (containing ≈ 50wt% curing agents, Ecoflex0030, Smooth-On, USA) and NdFeB microparticles (average diameter: 30 μm, Magnequench, China) with a weight ratio of 2:1 were uniformly mixed as a carrier. The magneto-active soft membrane was fabricated by spinning coating with a rotation speed of 500 r $min^{-1}$ and subsequently cured at 60 °C for 4 h. Copper nanoparticles were embedded in one surface of MRS to distinguish the top or bottom surface. The solidified magneto-active soft membrane was subsequently magnetized by a 3 T pulsed magnetic field using a magnetizer (MA-2030, Shenzhen JiuJu Company, China). The magneto-active soft membrane with a thickness of approximately 450 μm was cut into predesigned MRS patterns by focus laser beam using a pulse fiber laser carving machine (HAN's MUV-E-S12, China). The laser power was 2.1 W and the scanning speed was 800 mm $s^{-1}$.

### 1.2 Surface modification of MRS

SHP modification: a homemade superhydrophobic suspension, contained of 1 g fumed $SiO_2$ nanoparticles (average diameter of approximately 20 nm, Macklin, China) and 20 mL of normal hexane, was sprayed on the surface of MRS, and dried in air for 5 s. Janus-MRS modification: the surface coated with copper nanoparticles was sprayed with Nano-$SiO_2$ nanoparticles. LIS modification: the MRS was soaked in silicone oil with a viscosity of 360 mPa·s for 5 min, and the excess lubricant was removed by hanging the sample for 15 min.

**1.3 Characterization of MRS**

The morphologies of MRS, SHP-MRS and LIS-MRS were observed using a scanning electron microscope (SEM, JSM-6380LA, JEOL, Japan). The magnetic hysteresis loop of magneto-active soft membrane was measured using a vibrating sample magnetometer (VSM, Lake Shore 7410, USA). A magnetic indicator (Shanghai Daxue Electromagnetic Equipment Co., Ltd., China) was employed to visualize the magnetic field distribution and intensity of MRS. The mechanical performance of MRS was tested using a universal testing machine (LR10K Plus, Lloyd Instruments, Bognor Regis, UK) at a strain rate of 0.01 $s^{-1}$. The water contact angles were measured using a contact angle geometer (SL200B, KINO) at ambient temperature. The bounce behaviors of droplets were captured by a high-speed camera (960 fps, FASTEC-TS3, China). The optical images and videos of the switchable wettability on Janus-MRS, droplet transportation on SHP-MRS and droplet manipulation on LIS-MRS were recorded using an industrial digital camera (CM2000, KUY NICE, PEIPORT Group, Inc. China).

**1.4 Finite element analysis of magnetic actuation swing**

The swing process of MRS under magnetic actuation was analyzed using COMSOL Multiphysics (Version 5.4, COMSOL Inc., Sweden). The relative permeability of MRS and air were set as 1. The residual magnetization of microblades was set as 10.5 kA $m^{-1}$. The elastic modulus E and the Poisson's ratio of magneto-active soft membrane were 0.2 MPa and 0.49, respectively. The density of MRS was measured and set as 5.0 g $cm^{-3}$. The geometric model of the MRS was reconstructed. The swing process of MRS under magnetic actuation was analyzed in stationary and calculated in two segregated steps: magnetic force and rotation deformation. The model was meshed with tetrahedral in element size of “regular”. The magnetic field was set as 145 mT and rotated by 3.0° per step. The induced magnetic force was firstly calculated from the integral of the surface stress tensor on its boundaries. The deformation subjected to magnetic force was calculated by the solid mechanics module.

## 2. Supplementary Figures and Tables

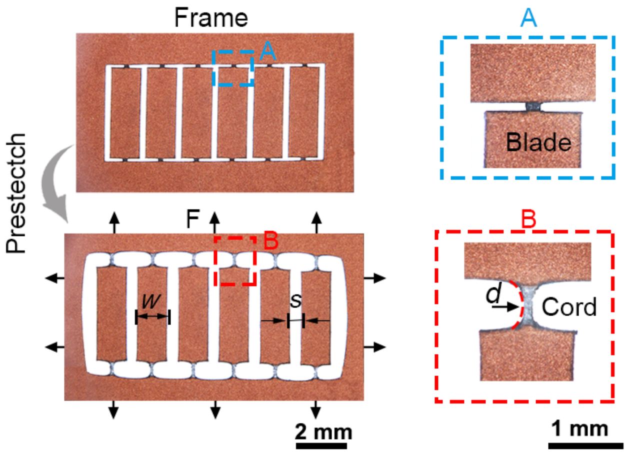


**Figure S1.** The morphology and parameters of the MRS before and after being pre-stretched. When MRS is in pre-stretched state, the distance between the adjacent microblades *s* becomes larger, the junction cords of the microblades turns slender with a diameter of *d*, and the width *w* of the microblades varies little.

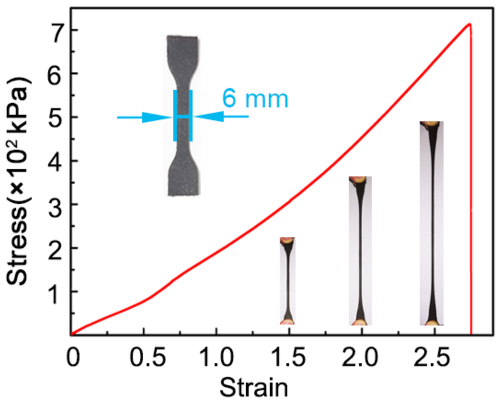


**Figure S2**. The stress-strain curves of magneto-active soft membrane under the maximum strain of 250%. The magnetoactive soft membrane exhibits excellent elasticity.

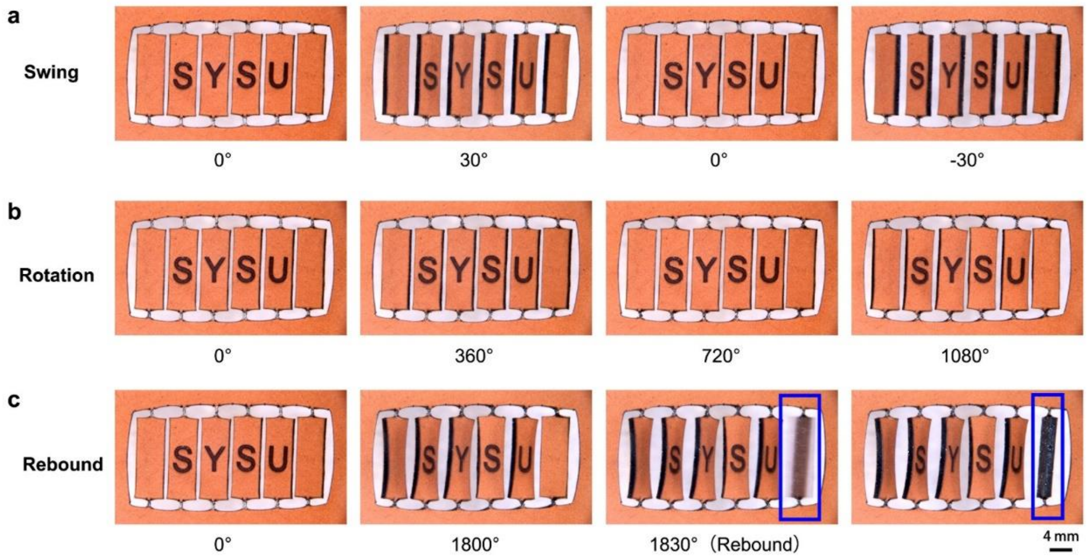


**Figure S3**. Transformation behaviors of MRS, including a) swing. b) rotation. c) The rebound of microblades is observed at the rotation angle of 1830°.

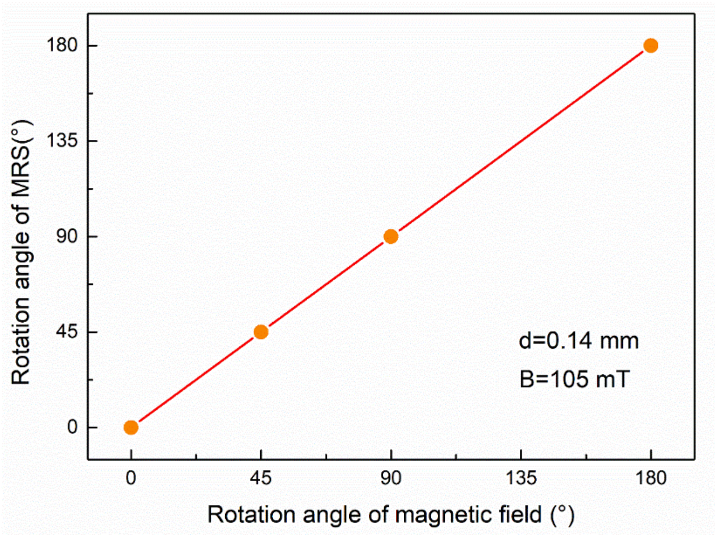


**Figure S4.** The rotation angle relationship between MRS and magnetic field. The rotation angles of the MRS can be accurately controlled by magnetic field B.

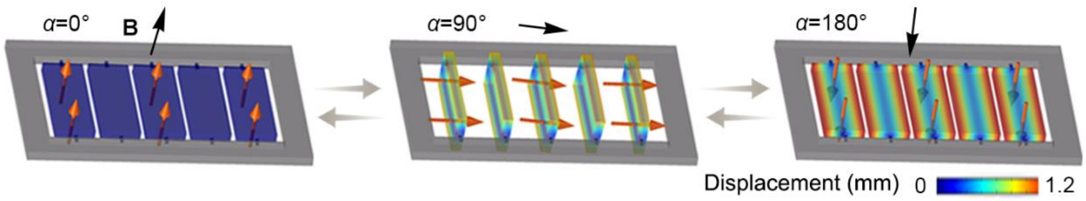


**Figure S5.** The finite element analysis of the rotation process of MRS under magnetic actuation. The simulated rotation process of MRS under magnetic actuation is well consistent with that of the experimental process.

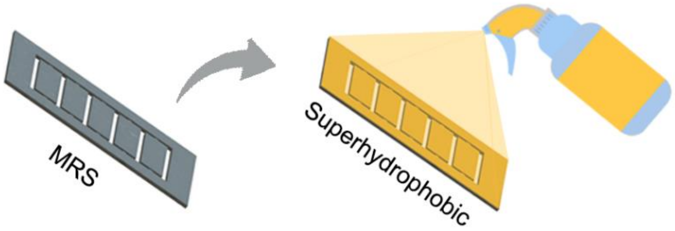


**Figure S6.** Spray treatment on the MRS. A facile superhydrophobic spray method is used to modify the surfaces of MRS. Specifically, the top surface of MRS was coated with a homemade superhydrophobic Nano-$SiO_2$ spray and the Janus-MRS was obtained. Both top and bottom surfaces of MRS were coated with the homemade superhydrophobic Nano-$SiO_2$ spray and the SHP-MRS was obtained.

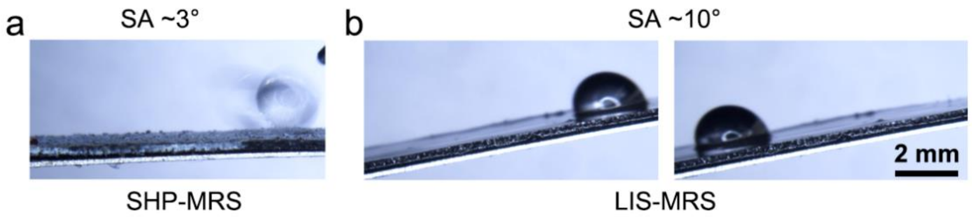


**Figure S7.** Sliding angle (SA) of a water droplet (~5 μL): a) SHP-MRS; b) LIS-MRS. SHP-MRS exhibits superhydrophobicity with teeny adhesion. LIS-MRS coated with silicone oil exhibits excellent slippery performance.

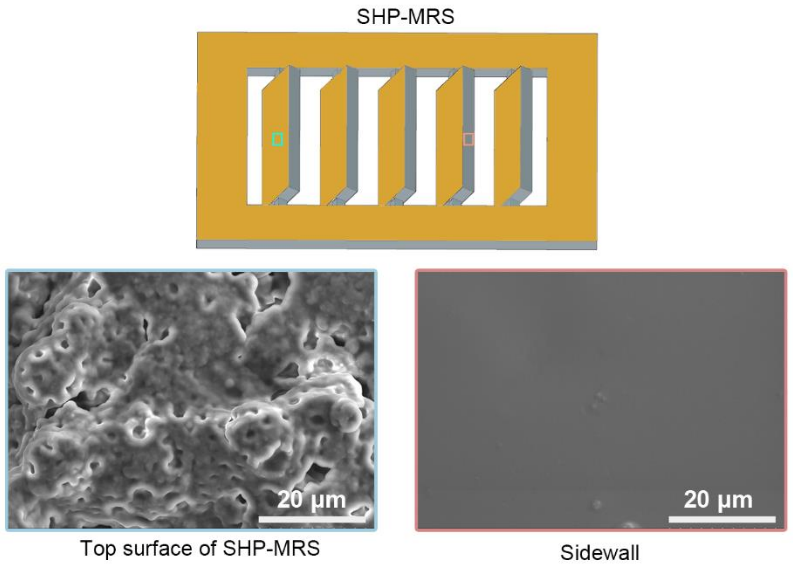


**Figure S8.** Schematic and SEM images of the surface and sidewall of SHP-MRS. The surface of the microblades is covered by superhydrophobic materials, and the sidewall remains exposed and has high adhesion to the water droplet.

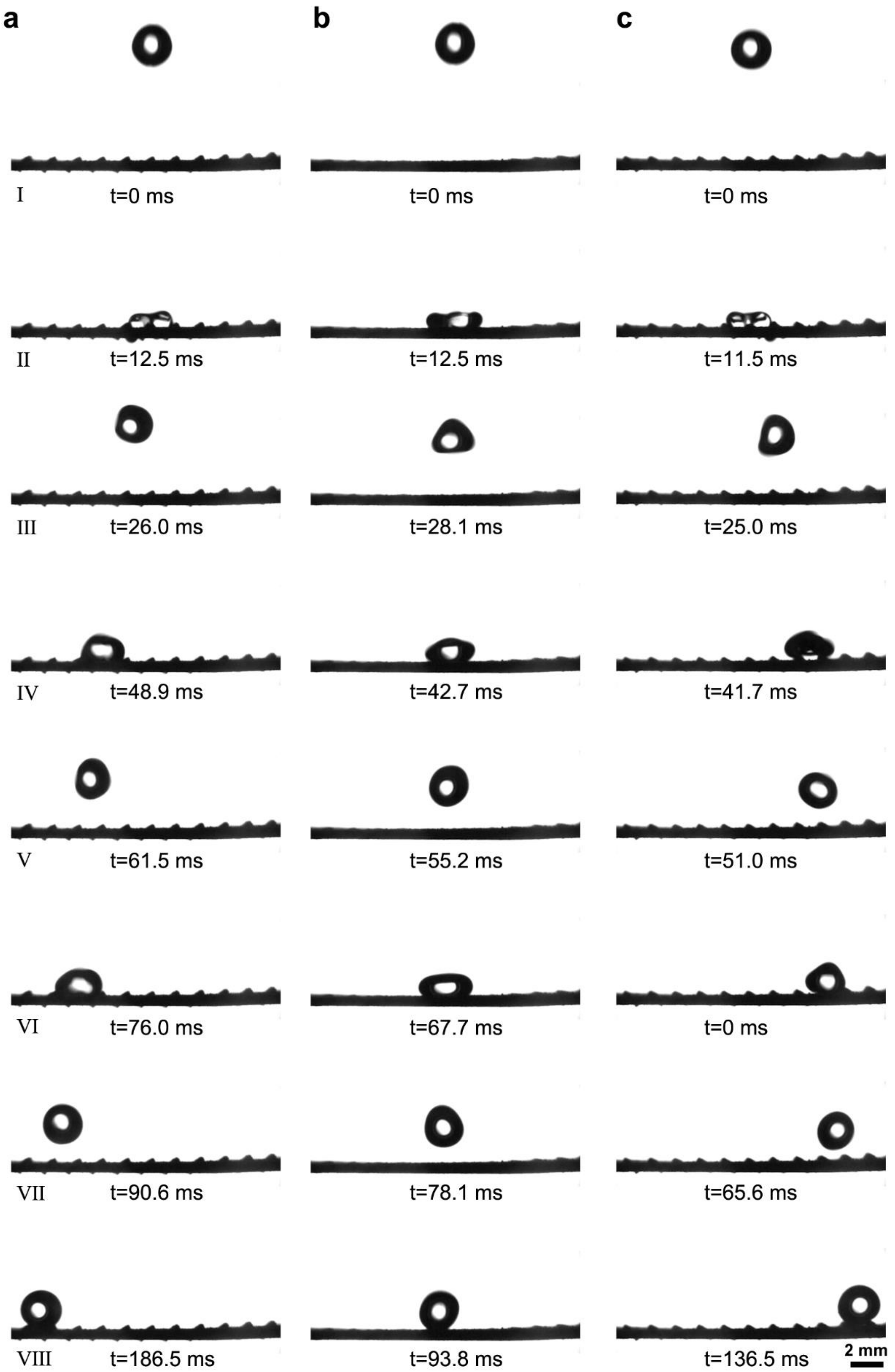


**Figure S9**. The detailed images of directional bounce behaviors of water droplets on the SHP-MRS with a) $\alpha$= -50°; b) $\alpha$= 0°; c) $\alpha$= 50°. When a droplet hits on the microblades at $\alpha$=0°, vertical pancake bounce is observed. When a droplet hits on the tilted microblades at α=-50° (50°), the droplet is flattened on the surface of microblades and then rebounded towards the left (right) due to the unbalanced rebound force (droplet-seesaw effect).

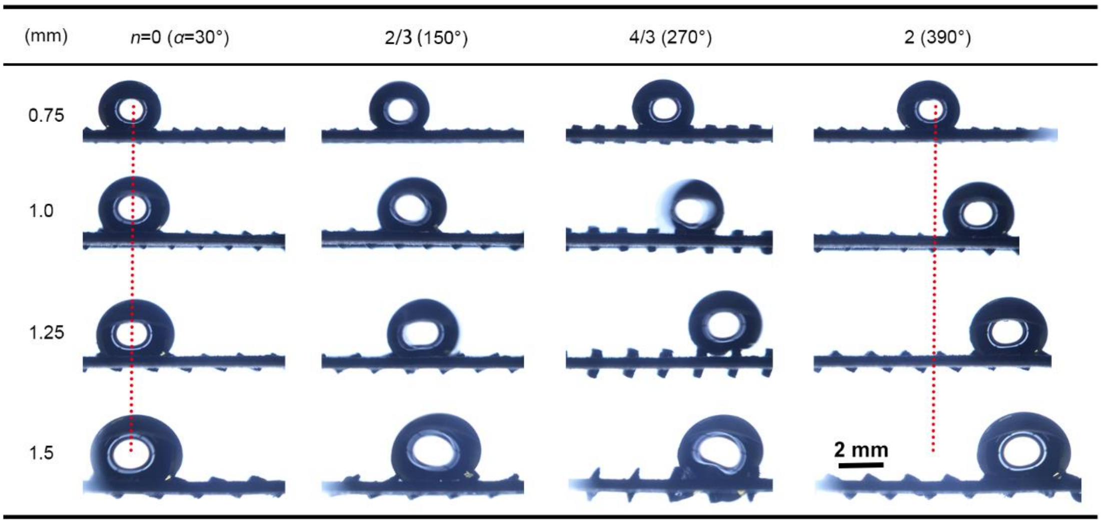


**Figure S10**. Droplet transport driven by microblade rotation of SHP-MRS with *w* of 0.75, 1.0, 1.25, and 1.5 mm. The droplet transport distance increases with the rotation angle $\alpha$ and the width *w* of the microblades.

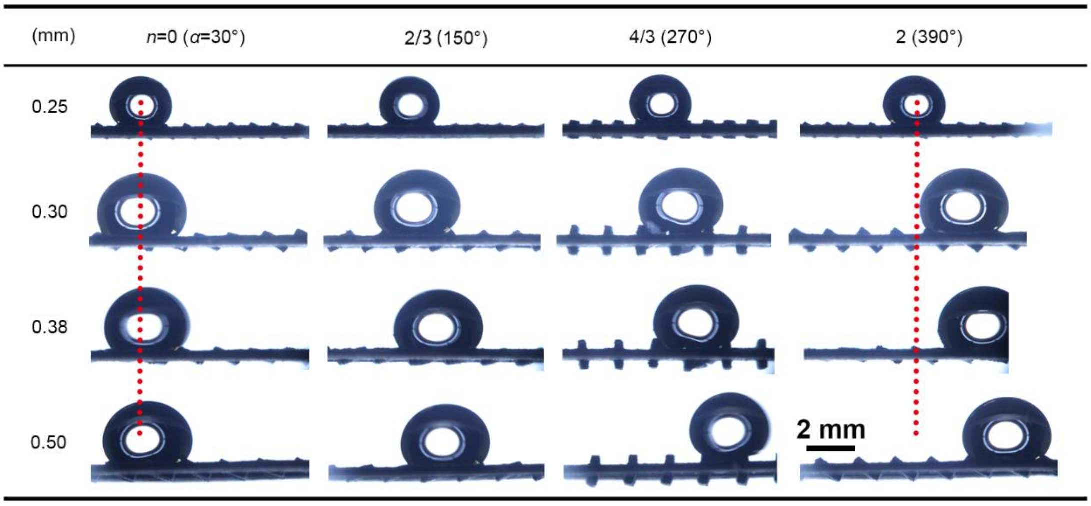


**Figure S11.** Droplet transport driven by microblade rotation of SHP-MRS with *s* of 0.25, 0.30, 0.38, and 1.50 mm. The droplet transport distance increases with the rotation angle $\alpha$ and the interval *s* of the microblades.

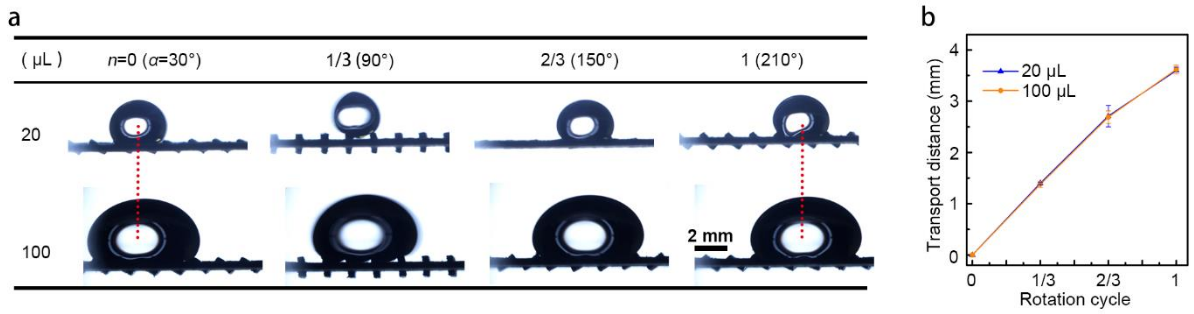


**Figure S12.** Droplet transport driven by microblade rotation of SHP-MRS with droplet volume of 20 and 100 µL: a) Optical images and b) Statistical chart. The droplet transport distance almost linearly increases with the rotation angle *α*. The droplet volume affects little on the transport distance.

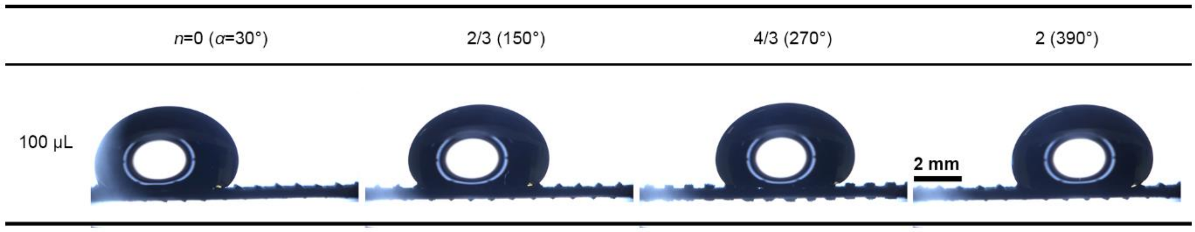


**Figure S13.** Ultra large droplet transport using SHP-MRS with *w*=0.75 mm and *s*=0.25 mm.

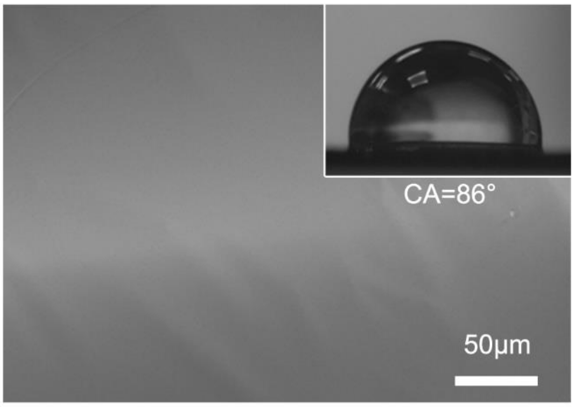


**Figure S14.** SEM image and water contact angle of LIS-MRS. The surface of LIS-MRS is smooth with a WCA of ≈86°.

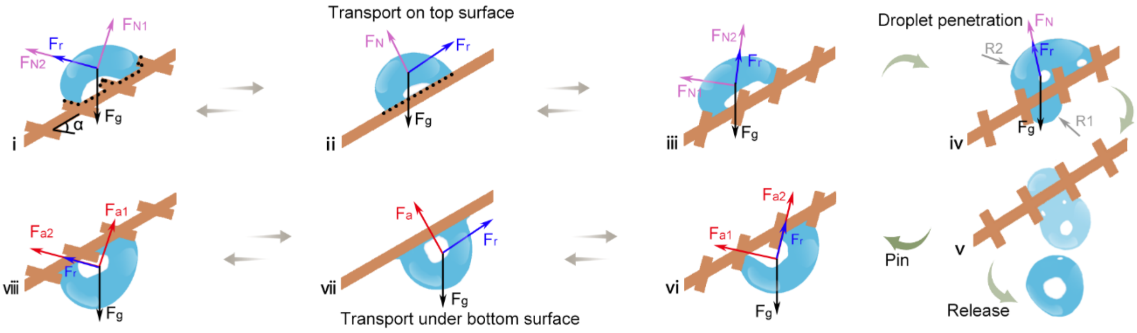


**Figure S15.** Schematic diagram of droplet transport on top surface, droplet penetration, and transport along the bottom surface of the LIS-MRS.

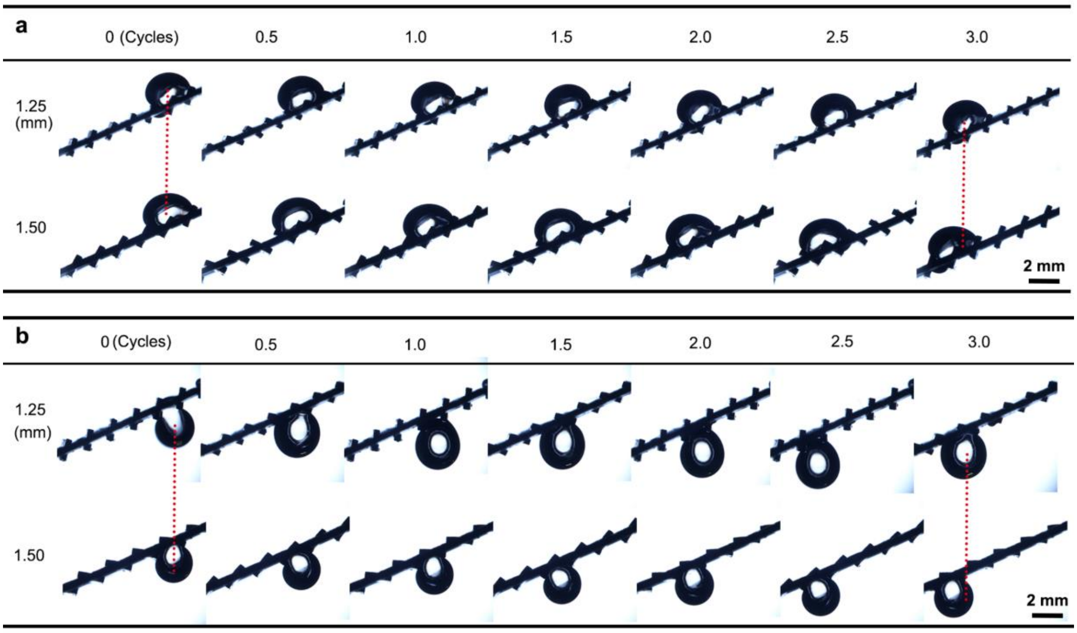


**Figure S16.** Droplet transport on the top surface of slant LIS-MRS with $w$ of 1.25 and 1.5 mm: a) top surface, and b) bottom surface. The droplet transport distance $l$ along the top surface or the bottom surface almost linearly increases with the swing cycles $n$ of actuating field, that is $l=n\ (w+s)$. The droplet transport distance $l$ also increases with the width $w$ of the microblades.

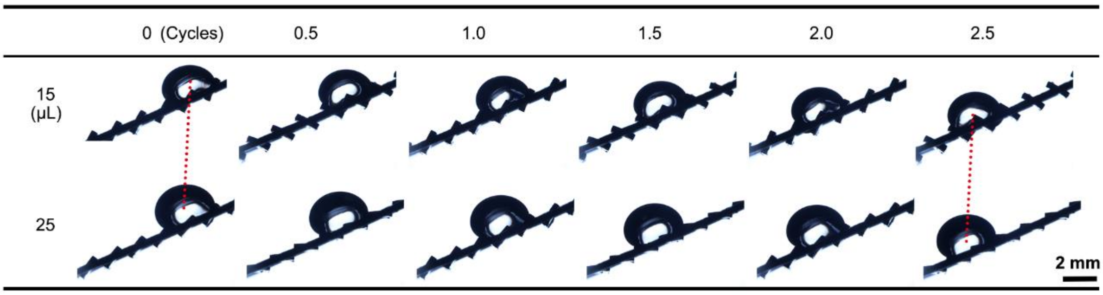


**Figure S17.** Droplet transport on the top surface of slant LIS-MRS with the droplet volume of 15 and 25 μL.

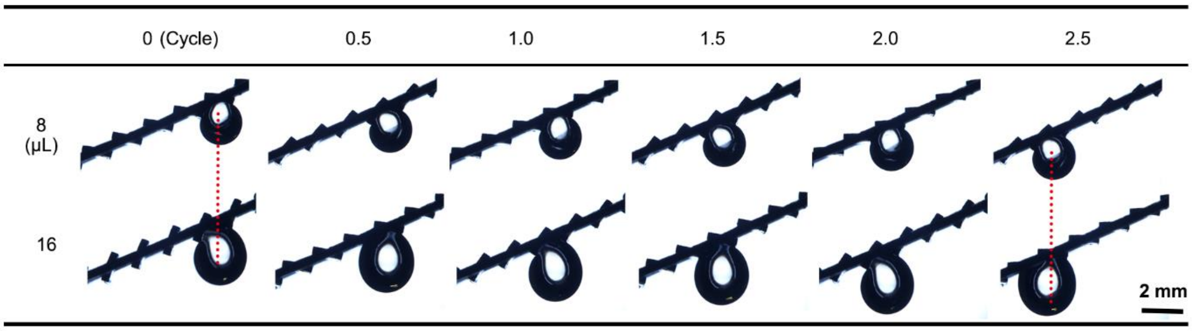


**Figure S18.** Droplet transport under the bottom surface of slant LIS-MRS with the droplet volume of 8 and 16 μL.

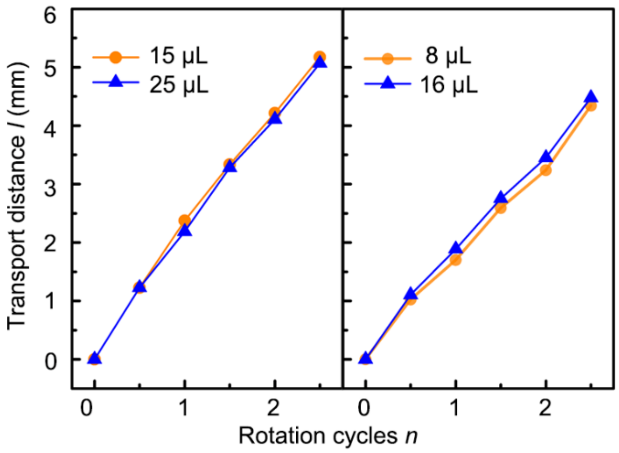


**Figure S19.** Droplet transport along the top surface and bottom surface of slant LIS-MRS with different droplet volumes: It can be clearly observed that the droplet volume affects little on transport distance both along top surface or bottom surface of slant LIS-MRS.

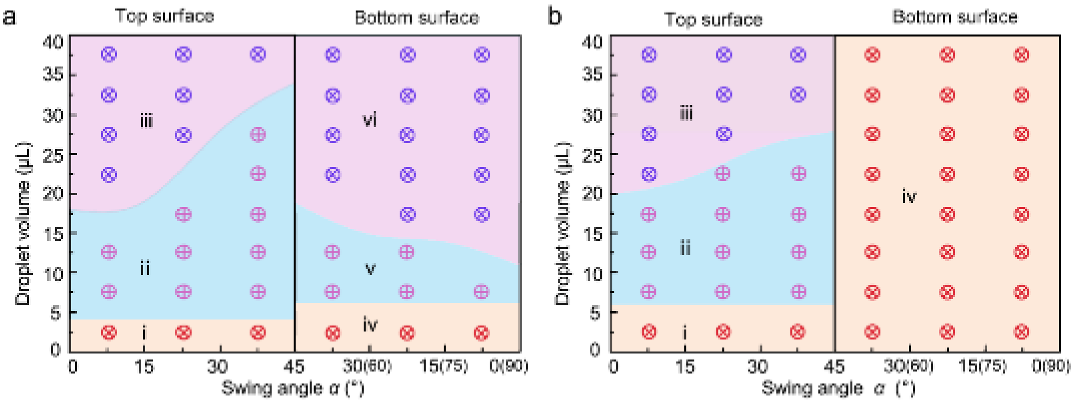


**Figure S20.** The phase diagram revealing droplet transport ability of slant LIS-MRS under different swing angles $\alpha$: a) The width of the microblades $w$ is 1.5 mm, and b) The width of the microblades $w$ is 1.0 mm. The regions **i** and **iv** represent that the small droplet cannot be transported along LIS-MRS. The regions **ii** and **v** represent that the droplet can be successfully transported along LIS-MRS. The regions **iii** and **vi** represent that the large droplet cannot be transported along LIS-MRS.  The droplet can't transport on the bottom

surface of the LIS-MRS with microblade width *w* of 1. 0 mm because the droplet can't penetrate the LIS-MRS due to large Laplace pressure.

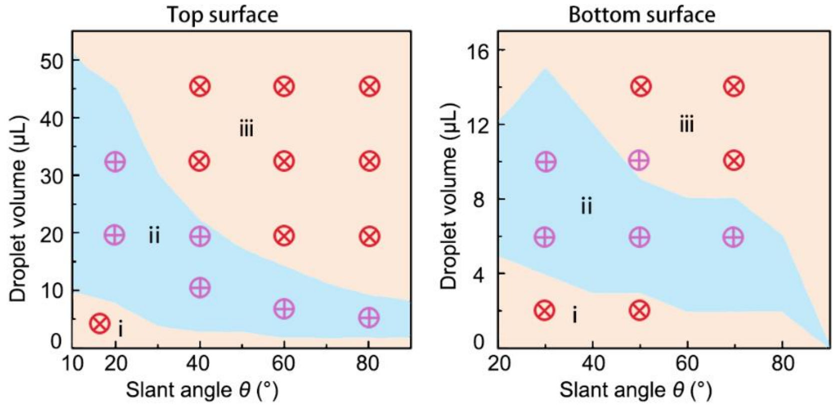


**Figure S21.** The volume ranges of droplet that can be correctly transported on the top (left) and bottom (right) surface of LIS-MRS. The regions **i** represents that small droplet cannot be transported along LIS-MRS. The regions **ii** represent that the droplet can be successfully transported along LIS-MRS. The regions **iii** represents that large droplet cannot be transported along LIS-MRS. The minimum slant angle $\theta$ required for droplet transport on top surface and bottom surface are about 10° and 20°, respectively. The volume ranges of the droplets, which can be successfully transported along the top and bottom surfaces, decrease with the slant angle $\theta$ of LIS-MRS. Because the gravity component on the droplet increases with slant angle $\theta$ of LIS-MRS, resulting in the droplet slipping down or falling off.

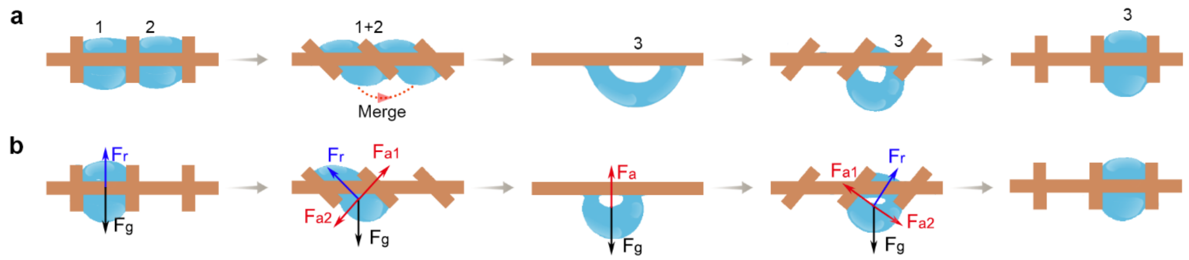


**Figure S22.** Schematic diagram and mechanism of droplets merging and droplet transport in the intervals of LIS-MRS.

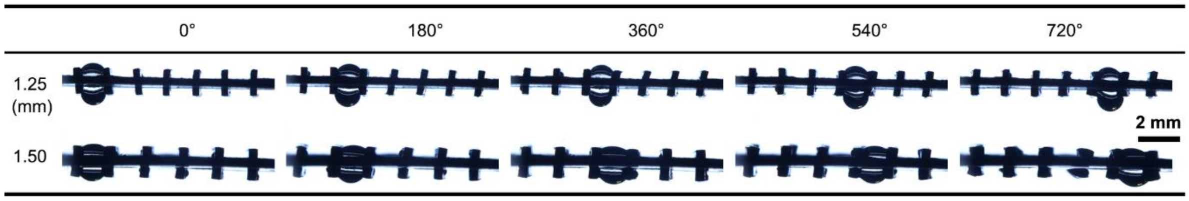


**Figure S23.** Droplet transport in the intervals of LIS-MRS with *w* of 1.25 and 1.5 mm. The droplet transport distance *l* increases with the microblade width *w*.

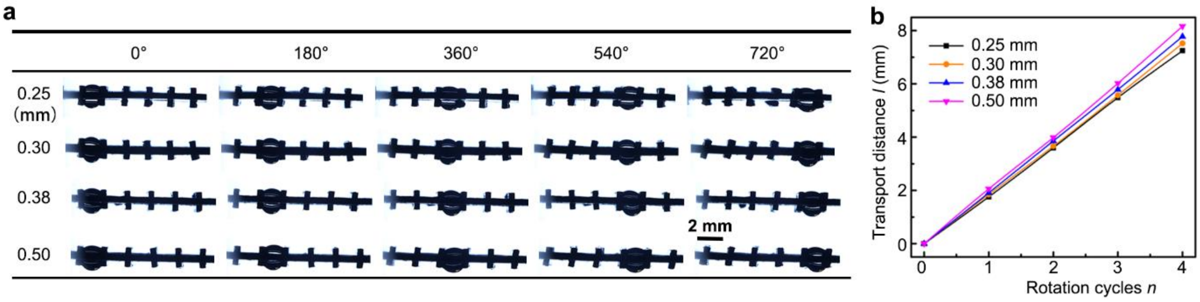


**Figure S24.** Droplet transport in the intervals of LIS-MRS with *s* of 0.25, 0.30, 0.38 and 0.50 mm: a) optical images, and b) statistical chart. The droplet transport distance *l* linearly increases with the rotation cycles *n*. The droplet transport distance *l* also increases with the microblade interval *s*.

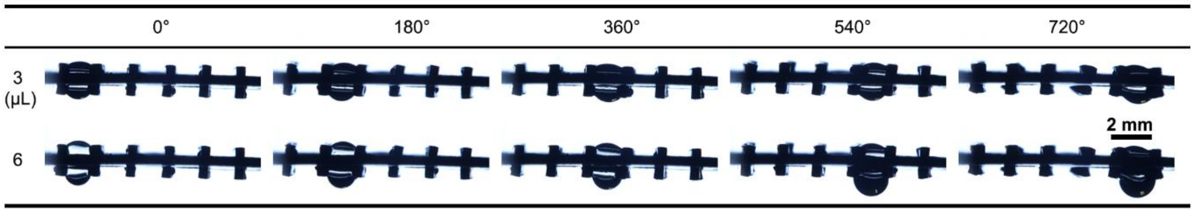


**Figure S25.** The droplet transport in the intervals of LIS-MRS under different droplet volumes of 3 and 6 μL. The droplet volume affects little on droplet transport distance *l*.

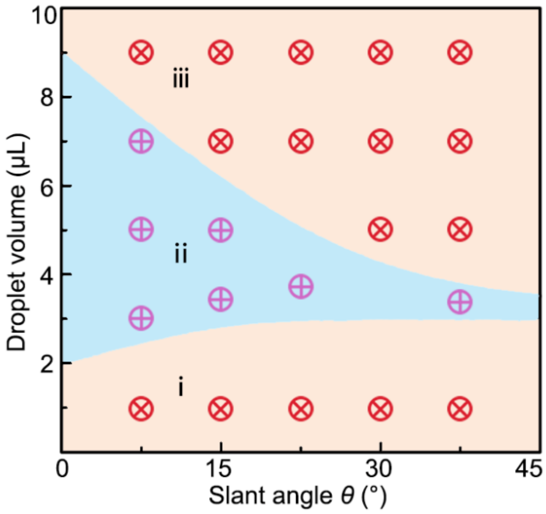


**Figure S26.** The anti-gravity ability of droplet transport in the intervals of LIS-MRS. The region i represents that small droplet cannot be transported along LIS-MRS. The region ii represents that the droplet can be successfully transported along LIS-MRS. The region iii represents that large droplet cannot be transported along LIS-MRS. The volumes of transport droplet that is successfully transported in the intervals of microblades decrease with the slant angle $\theta$ of LIS-MRS.

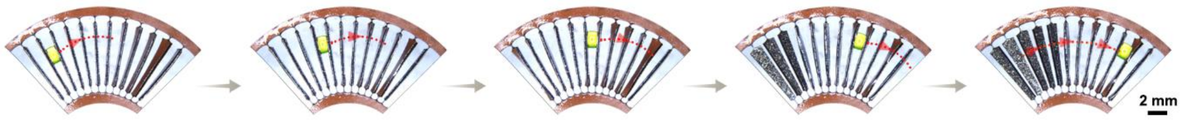


**Figure S27.** Droplet transport in the intervals of fan-shaped LIS-MRS. The droplet also can be transported in a particular arc path using a fan-shaped LIS-MRS, further manifesting the droplet transport ability.

**Table S1**. The detailed geometric parameters of MRS.

| | | | | | | |
|---|---|---|---|---|---|---|
| *d* (mm) | 0.07 | 0.14 | 0.20 | 0.35 | 0.47 | 0.68 |
| *w* (mm) | 0.75 | 1.0 | 1.25 | 1.5 | | |
| *s* (mm) | 0.25 | 0.30 | 0.38 | 0.50 | | |

## 3. Supplementary Discussion

### 3.1 Droplet transport on the top surface

As shown in Figure 4d and Figure S14 (**i**, **ii** and **iii**), (I) When the swing angle of magnetic field is at $\alpha$ (0°<$\alpha$<45°), the droplet is pinned on the microblades 1, 2 and 3 (Figure S14-**i**). There exists the gravitational force ($\mathbf{F}_g$), surface retention force ($\mathbf{F}_r$) and positive direction support force ($\mathbf{F}_N$). $\mathbf{F}_N$ is the resultant force of $\mathbf{F}_{N1}$ (surfaces of microblades) and $\mathbf{F}_{N2}$ (sidewalls of microblades). $\mathbf{F}_g$ is equal to the resultant of $\mathbf{F}_N$ and $\mathbf{F}_r$. (II) When the microblades are clockwise swung to α=0°, resulting in an unstable state of droplet due to the rapid decrease of the triple-phase contact line (TPCL). The droplet detached from microblade 1 and then moves to the microblades 2&3. (Figure S14-**ii**). There exists the gravitational force ($\mathbf{F}_g$), surface retention force ($\mathbf{F}_r$) and positive direction force ($\mathbf{F}_N$). $\mathbf{F}_g$ is equal to the resultant of $\mathbf{F}_N$ and $\mathbf{F}_r$. (III) When the microblades are continuously clockwise rotated with -45° < α < 0°, the droplet slips down and is pined on the microblades 2, 3 and 4, tending to a more stable state of droplet due to the increase of triple-phase contact line and release of surface energy (Figure S14-**iii**). There exists the gravitational force ($\mathbf{F}_g$), surface retention force ($\mathbf{F}_r$) and positive direction force ($\mathbf{F}_N$). $\mathbf{F}_N$ is the resultant force of $\mathbf{F}_{N1}$ (surfaces of microblades) and $\mathbf{F}_{N2}$ (sidewalls of microblades). $\mathbf{F}_g$ is equal to the resultant of $\mathbf{F}_N$ and $\mathbf{F}_r$.

### 3.2 Droplet transport under the bottom surface

As shown in Figure 4e and Figure S14 (**vi**, **vii** and **viii**), (I) When the swing angle of magnetic field is $\alpha$ (0°<$\alpha$<45°), the droplet is pinned on the microblades 1, 2 and 3. (Figure S14-**vi**). There exists the gravitational force ($\mathbf{F}_g$), surface retention force ($\mathbf{F}_r$) and adhesion force ($\mathbf{F}_a$). $\mathbf{F}_a$ is the resultant force of $\mathbf{F}_{a1}$ (surfaces of microblades) and $\mathbf{F}_{a2}$ (sidewalls of microblades). $\mathbf{F}_g$ is equal to the resultant of $\mathbf{F}_a$ and $\mathbf{F}_r$. (II) When the microblades are clockwise rotated to $\alpha$=0°, resulting in an unstable state of droplet due to the rapid decrease of the triple-phase contact line (TPCL). The droplet detached from microblade 1 and then moves to the microblades 2&3. (Figure S14-**vii**). There exists the gravitational force ($\mathbf{F}_g$), surface retention force ($\mathbf{F}_r$) and adhesion force ($\mathbf{F}_a$). $\mathbf{F}_g$ is equal to the resultant of $\mathbf{F}_a$ and $\mathbf{F}_r$. (III) When the microblades are continuously clockwise rotated at -45° < α < 0°, the droplet slips down and is pined on the microblades 2, 3 and 4, tending to a more stable state of droplet due to the increase of triple-phase contact line and release of surface energy (Figure S14-**viii**). The gravitational force ($\mathbf{F}_g$), surface retention force ($\mathbf{F}_r$) and adhesion force ($\mathbf{F}_a$) existed. $\mathbf{F}_a$ is the resultant force of $\mathbf{F}_{a1}$ (surfaces of microblades) and $\mathbf{F}_{a2}$ (sidewalls of microblades). $\mathbf{F}_g$ is equal to the resultant of $\mathbf{F}_a$ and $\mathbf{F}_r$.

### 3.3 Droplet directionally penetrate from the interval of the microblades

As shown in Figure S14, the droplet can penetrate through the interval of the microblades once the swing angle $\alpha$ beyond 45°. There exists the gravitational force ($\mathbf{F}_g$), surface retention force ($\mathbf{F}_r$), support force ($\mathbf{F}_N$) and Laplace pressure different (△**P**). $\mathbf{F}_g$ facilitates the droplet penetrate through the interval of the microblades while △**P** and the resultant force of $\mathbf{F}_r$ and $\mathbf{F}_N$ prevent the droplet penetration. △**P** enhances with the interval *s* of the microblades. The directional penetration ability of droplet enhances with the droplet volume and the interval *s* of the microblades. Once the droplet penetrates through the interval of the microblades, the droplet may get stuck by the bottom surface or fall off from LIS-MRS. The droplet get stuck by the bottom surface when $\mathbf{F}_g$ is smaller than the resultant force of $\mathbf{F}_r$

and $\mathbf{F}_a$. The droplet falls off from LIS-MRS when $\mathbf{F}_g$ is smaller than the resultant force of $\mathbf{F}_r$ and $\mathbf{F}_a$.

**3.4 Droplets merging and droplet transport in the intervals of LIS-MRS**

To further understand the mechanism of the magnetically responsive droplets merging and droplet transport in the intervals of LIS-MRS, the forces on the droplets were analyzed (Supplementary Figure S21). (I) Two droplets (droplet 1 and droplet 2) are sandwiched in the adjacent intervals of microblades. There exists the gravitational force ($\mathbf{F}_g$) and the surface retention force ($\mathbf{F}_r$) and $\mathbf{F}_g = \mathbf{F}_r$. (II) When the microblades are anticlockwise rotated, the droplets are gradually extruded by the microblades. Two extruded droplets contact with each other and are merged into a bigger droplet (droplet 3) to lower the surface tension. There exisits gravitational force ($\mathbf{F}_g$), the surface retention force ($\mathbf{F}_r$) and the adhesion force ($\mathbf{F}_a$, induced from the microblades). $\mathbf{F}_a$ is the resultant force of $\mathbf{F}_{a1}$ (right microblade) and $\mathbf{F}_{a2}$ (left microblade). $\mathbf{F}_g$ is equal to the resultant of $\mathbf{F}_a$ and $\mathbf{F}_r$. (III) When the microblades are rotated at $\alpha$=0°, droplet 3 hangs by the bottom surface of microblades under the combination of gravity and adhesion. There exists the gravitational force ($\mathbf{F}_g$) and the adhesion force ($\mathbf{F}_a$) and $\mathbf{F}_g = \mathbf{F}_a$. (IV) When the microblades are continuously rotated, the droplet is transferred to contact with the third microblade. (V) When the microblades were rotated at α = 90°, the droplet is sandwiched between the second and third microblades due to the release of surface energy. In brief, the contact area between the droplet and the microblades is decisive and related to the total surface energy of the droplet. When the droplet is sandwiched into the adjacent microblade, the contact area is the largest with the most stable state.

**4. Supplementary Videos**

**Video S1**. Transformation behaviors of MRS under magnetic actuation, including reversible swing and rotation. The microblades can achieve two manipulation modes including reversible swing and rotation. Swing mode: the microblades swing back and forth under a

pendular actuating field. Rotation mode: the rotating magnetic field synchronously actuates the microblades continuous rotation for several circles.

**Video S2**. Experimental and simulated rotation process of MRS under magnetic actuation. The simulated rotation process of MRS under magnetic actuation is well consistent with that of the experimental process. The microblades synchronously rotate for approximately 180° with clockwise rotating magnetic field **B**.

**Video S3**. Switchable wettability of Janus-MRS. When the top surface is facing up, the water droplet spontaneously forms into spherical shape in superhydrophobic state on the top surface. Correspondingly, when the bottom surface is facing up, the water droplet is pinned in hydrophilic state on the bottom surface.

**Video S4**. Jump behavior of water droplets on the Janus-MRS. When the top surface is turned to the front side under magnetic actuation, the water droplet is firstly impacted on the superhydrophobic surface and then bounced up to the air in 14.3 ms. However, when the bottom surface is swung to the front side under magnetic actuation, the water droplet falls down and is immediately pinned on the hydrophilic surface.

**Video S5**. Bounce behaviors of water droplets on the surface of SHP-MRS. When a droplet falls down and hits on the microblades with $\alpha$=0°, vertical pancake bounce can be found on the surface. When the droplet hits the tilted microblades with $\alpha$=50° (-50°), the droplet is flattened on the surface and then rebounded towards the left (right) attributed to the unbalanced rebound force induced by the tilted microblades.

**Video S6**. Controllable droplet transport along SHP-MRS. SHP-MRS can precisely transport droplet interval-by-interval driven by rotating magnetic field. The droplet can move back and forth by switching the direction of rotating magnetic field. SHP-MRS is able to transport ultra-large droplet (≥100 μL).

**Video S7**. Droplet transport along a fan-shape SHP-MRS and simple chemical reaction conducted by SHP-MRS. The fan-shaped SHP-MRS delivers a droplet in a particular arc path. SHP-MRS is used as a miniature reactor for rapid microchemical reaction.

**Video S8**. Continuous droplet transport process, composed of transport along upper surface, unidirectional penetration, and transportation along lower surface. The water droplet is delivered on the top surface interval-by-interval via periodical swing of the microblades of slant LIS-MRS. When this droplet is transferred to a target position, it can penetrate through the interval between microblades by increasing swing angle. Subsequently, the penetrated droplet is hanged by the microblades and transported along the bottom surface via periodical swing of magnetic field.

**Video S9**. Magnetically responsive chemical reaction platform based on LIS-MRS. One by one, three droplets (NaOH, NaOH, and HCl) are accurately transported to the target positions, penetrates through the appropriate intervals of microblades, and are released to react with the underlying droplets (pp, CuSO4, and pp+NaOH).

**Video S10**. Droplets merge and droplet transport in the intervals of LIS-MRS. Two adjacent droplets are merged as the microblades are continuously rotated. The merged droplet can be transported from one interval to the next interval by anticlockwise rotation of the microblades.

**Video S11**. Droplets merging and droplet transport on a horizontal, slant, curved and fan-shaped LIS-MRS. Droplet 1 and 2 are firstly merged into droplet 3. Droplet 3 moves back and forth along the horizontal, slant, curved and fan-shaped LIS-MRS under magnetic actuation.

**Video S12**. Transport of droplet array in the intervals of LIS-MRS. The droplet array (2×3) is transferred forth and back interval by interval using the LIS-MRS.
